# Evidence against the energetic cost hypothesis for the short introns in highly expressed genes

**DOI:** 10.1186/1471-2148-8-154

**Published:** 2008-05-20

**Authors:** Yi-Fei Huang, Deng-Ke Niu

**Affiliations:** 1 MOE Key Laboratory for Biodiversity Science and Ecological Engineering, College of Life Sciences, Beijing Normal University, Beijing 100875, P R China

## Abstract

**Background:**

In animals, the moss *Physcomitrella patens *and the pollen of *Arabidopsis thaliana*, highly expressed genes have shorter introns than weakly expressed genes. A popular explanation for this is selection for transcription efficiency, which includes two sub-hypotheses: to minimize the energetic cost or to minimize the time cost.

**Results:**

In an individual human, different organs may differ up to hundreds of times in cell number (for example, a liver versus a hypothalamus). Considered at the individual level, a gene specifically expressed in a large organ is actually transcribed tens or hundreds of times more than a gene with a similar expression level (a measure of mRNA abundance per cell) specifically expressed in a small organ. According to the energetic cost hypothesis, the former should have shorter introns than the latter. However, in humans and mice we have not found significant differences in intron length between large-tissue/organ-specific genes and small-tissue/organ-specific genes with similar expression levels. Qualitative estimation shows that the deleterious effect (that is, the energetic burden) of long introns in highly expressed genes is too negligible to be efficiently selected against in mammals.

**Conclusion:**

The short introns in highly expressed genes should not be attributed to energy constraint. We evaluated evidence for the time cost hypothesis and other alternatives.

## Background

In animals (including humans, mice and *Caenorhabditis elegans*), the moss *Physcomitrella patens *and the pollen of *Arabidopsis thaliana*, highly expressed genes have been found to have short introns and exons [1-7]. Several hypotheses have been proposed to explain the compactness of highly expressed genes. The first, based on the fact that transcription is a slow and expensive process, suggests that natural selection for transcriptional efficiency favors the compactness of highly expressed genes [1,8,9]. The second hypothesis, called "genome design", suggests that highly expressed genes are short because most of them are housekeeping genes whose epigenetic regulation is less complex than that of weakly expressed tissue-specific genes [10]. In line with this hypothesis, expression level and breadth are strongly positively correlated, and human housekeeping genes are more compact than tissue-specific genes [9,10]. However, by comparing artificially selected pairs of housekeeping and narrowly expressed genes with similar average expression levels, Li et al. [6] recently found that housekeeping genes are no more compact than narrowly expressed genes if the expression level is controlled. This implies that expression level rather than breadth determines the compactness of genes. The third hypothesis is mutational bias, which supposes that highly expressed genes tend to localize in chromosomal regions with high deletion rates, or that there is a transcription-associated deletion bias [2,5]. Urrutia and Hurst [5] found that the introns of highly expressed genes are still small even if the effects of chromosomal regions are controlled. Housekeeping genes are expected to have much higher germline transcriptional frequencies, and thus, more transcription-associated deletions, than genes that are narrowly expressed in somatic tissues. However, Li et al [6] found that housekeeping genes are no more compact than genes that are narrowly expressed in somatic tissues with similar average expression levels.

The transcription efficiency hypothesis includes two sub-hypotheses: an energetic cost hypothesis and a time cost hypothesis. Selection for short introns and short exons may be driven either by minimizing the energetic cost of transcription or by the requirement to transcribe large amounts of mRNA molecules within limited periods. Human antisense genes that have very short response times have been found to have short introns [11,12], which directly supports the time cost hypothesis. Furthermore, Jeffares et al. [13] found that the intron density in common eukaryotes is positively correlated with the duration of life cycle. However, the time cost hypothesis has been argued against or overlooked in recent studies [3,4,6]. Seoighe et al. [3] pointed out that the transcription of multiple copies of mRNA does not necessarily require a much longer period of time than required to transcribe the first copy, because multiple polymerases may be simultaneously working on one template [14]. The present paper presents evidence against the energetic cost hypothesis and evaluates evidence for the time cost hypothesis and other alternatives.

In animals, different organs may differ up to hundreds of times in cell number and weight. For example, in an adult human, a lung weighs about 1000 g while a prostate weighs only about 20 g. Thus, humans produce tens of times more mRNA molecules for a lung-specific gene (for example, *SFTPD*) than for a prostate-specific gene (for example, *SEMG1*) with a similar expression level (considered to be a measure of mRNA abundance per cell in this paper; see Methods for the source of the expression data of these two example genes). Expression of *SFTPD *is thus expected to have tens of times higher energetic cost to a human body than expression of *SEMG1*, if these two genes have similar lengths. According to the energetic cost hypothesis, *SFTPD *should have much shorter introns than *SEMG1*. On the contrary, *SFTPD *has a longer average intron length and total intron length than *SEMG1 *(Additional File 1). The present paper surveys large-tissue/organ-specific (LTS) genes and small-tissue/organ-specific (STS) genes at a genome-wide scale and compares their compactness for a statistically convincing result.

## Results and Discussion

### Large-tissue/organ-specific genes and small-tissue/organ-specific genes have similar sizes

The gene expression datasets we used include the gene expression levels in 69 non-disease adult tissue/organ samples from humans and 55 non-disease adult tissue/organ samples from mice [15]. The weights of these tissue/organs are on a continuum varying by several magnitudes. For reliability, only the largest samples are defined as large tissue/organs and the smallest samples are defined as small tissue/organs (Table 1). The sizes of tissue/organs were determined by searching the literature [16-24] and internet resources (for example, Wikipedia, the free encyclopedia), or estimated by experience. A conservative estimation of the difference in average tissue/organ weight between large tissue/organ samples and small tissue/organ samples is > 50 times.

**Table 1:** Tissue/organ samples and the number of specific genes analyzed in this study^a^

	Large tissue/organ (number of specific genes; tissue/organ weight)^b^	Small tissue/organ (number of specific genes; tissue/organ weight)^b^
*Homo sapiens*	Cultured adipocytes (18; 9 Kg)	Brain amygdala (22; --)
	Liver (79; 1.5 Kg)	Hypothalamus (7; 4 g)
	Lung (18; 1 Kg)	Pituitary (6; 5 g)
	Skeletal muscle (4; 27 Kg)	Tonsil (1; 30–40 g)
	Skin (6; 5 Kg)	Prostate (13; 20 g)
	Smooth muscle (24; --)	Thymus (11; 30–40 g)
		Thyroid (25; 18–60 g)
		Tongue (11; 70 g)
*Mus musculus*	Adipose tissue (13; --)	Amygdala (4; --)
	Liver (76; 2 g)	Hypothalamus (12; < 60 mg)
	Skeletal muscle (47; --)	Pituitary (29; 3 mg)
	Epidermis (4; --)	Trigeminal (7; --)
		Prostate (24; 0.11 g)
		Thymus (64; < 60 mg)
		Thyroid (21; 15 mg)
		Tongue epidermis (14; --)
		Retina (71; --)

^a^See Additional File 1 and Additional File 2 for full lists of these genes.

^b ^Some samples, like the subthalamic nucleus and trigeminal ganglion, are undoubtedly small tissues/organs. These may be not included in this study because we could not find any specific genes for them. The tissue/organ weights were obtained directly from literatures and internet resources (for example, Wikipedia, the free encyclopedia) or calculated according to their ratio to body by assuming that the weights of adult human and mouse bodies are about 70 Kg and 30 g, respectively (when different sources of data are not consistent, we retained the conservative estimation) [16–24, 34]. Some samples (like smooth muscle, tongue epidermis and retina) were categorized into large tissue/organs or small tissue/organs on the basis of experience. Some mouse tissue/organs were categorized by consulting their human homologs. In humans, the lower limit of large tissue/organ samples was lung (about 1000 g), while the upper limit of small tissue/organ samples was tongue (70 g). In mice, the lower limit of large tissue/organ samples was liver (about 2 g), while the upper limit of small tissue/organ samples was prostate (0.11 g).

**Figure 1:**
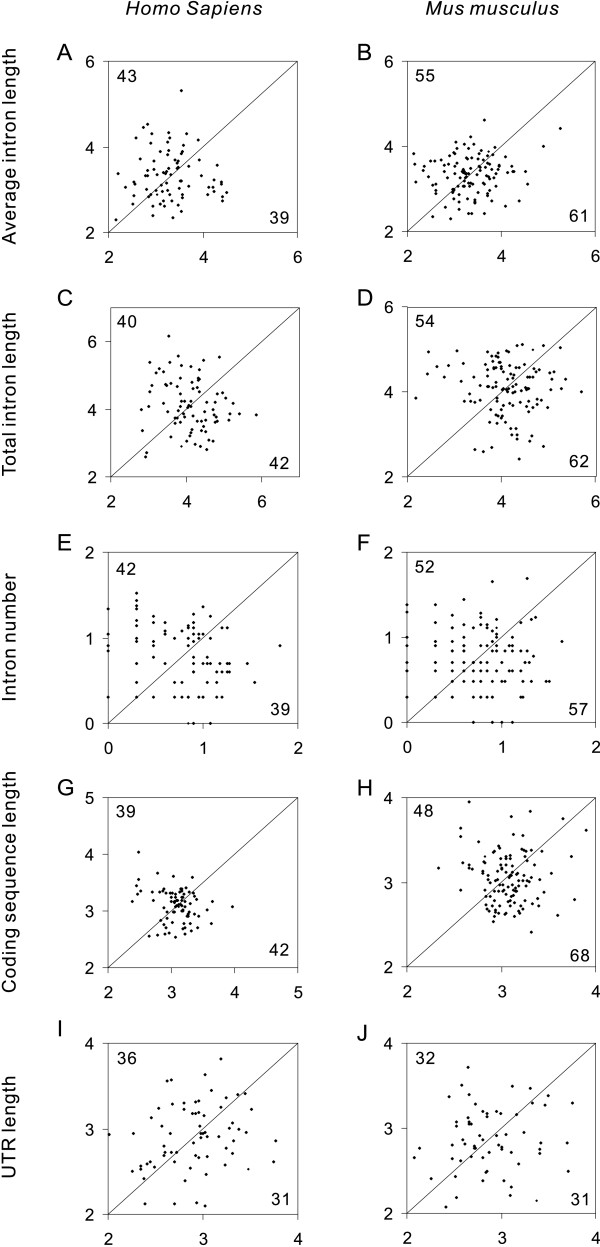
**Comparison of large-tissue/organ-specific genes and small-tissue/organ-specific genes with similar expression levels.** The logarithm (base 10) values are shown. The Y axis represents small-tissue/organ-specific genes, while the X axis shows their large-tissue/organ-specific counterparts. The numbers of dots above (marked at the top left corner) and below (marked at the bottom right corner) the diagonal line illustrate the comparison between large-tissue/organ-specific genes and small-tissue/organ-specific genes. We performed Wilcoxon signed ranks tests to determine the significance of the differences. The number of gene pairs and the significance levels are: (A) 82, *P *= 0.59; (B) 116, *P *= 0.39; (C) 82, *P *= 0.57; (D) 116, *P *= 0.81; (E) 82, *P *= 0.90; (F) 116, *P *= 0.57; (G) 82, *P *= 0.86; (H) 116, *P *= 0.50; (I) 67, *P *= 0.89; (J) 63, *P *= 0.83.

Tissue/organ-specific genes are those that are expressed only in one particular tissue/organ sample. In total, we found 149 LTS genes and 96 STS genes in humans and 140 LTS genes and 246 STS genes in mice (Table 1, Additional Files 1, 2). As the tissue/organ weights differed by tens or even hundreds of times, an LTS gene is expected to produce tens or even hundreds of times more mRNA molecules per tissue/organ than an STS gene with a similar expression level. If the compactness of highly expressed genes has evolved to minimize the energetic cost of transcription, the LTS genes should be more compact than the STS genes with similar expression levels. However, pairwise comparisons of LTS-STS gene pairs with similar expression levels (for details, see Methods) do not show significant differences in average intron length, total intron length, intron number, coding sequence (CDS) length or untranslated region (UTR) length between LTS genes and STS genes, in either humans or mice (Figure 1).

How large a difference in expression level is required to generate a significant difference in gene compactness? The genes analyzed above were divided on the basis of expression level, rather than the size of tissue/organ; genes in the top 30% quantile were considered to be highly expressed and those in the bottom 30% quantile were considered to be weakly expressed genes. As shown in Table 2, the introns and UTRs of highly expressed genes are significantly shorter than those of weakly expressed genes, but there is no significant difference in intron number or CDS length (Table 2). This result is in contrast to a previous study [6], but is in line with another study, which found that total exon length is much more weakly related to expression level than intron length [1]. We suspect that the small number of genes analyzed in this study may have obscured a weak trend. One might expect that increasing the difference in expression level between highly expressed and weakly expressed genes (for example comparing genes in the top 10% quantile with those in the bottom 10% quantile) would reveal significant differences in intron number and CDS length. In fact, selecting 10% quantiles resulted in a much smaller number of genes being analyzed and, consequently, statistically less convincing results (data not shown). The difference in expression level between the top and bottom 30% quantiles of human genes or mouse genes is about 20 times (Table 2). As the expression value detected by microarray is linear with the concentration of target RNA (Affymetrix 2001, technical note, new statistical algorithms for monitoring gene expression on GeneChip^® ^probe arrays), this difference in expression level can reflect the difference in the concentrations of the target mRNAs.

**Table 2:** Comparison of compactness between genes expressed at different levels^a^

	Average intron length	Total intron length	Intron number	CDS length	UTR length	Expression level
Human genes						
Top 30% quantile	2768 ± 608	28117 ± 7347	8 ± 1	1313 ± 90	775 ± 107	5369 ± 770
Versus						
Bottom 30% quantile	10448 ± 4237	901046 ± 33210	9 ± 1	1764 ± 232	1478 ± 244	267 ± 14
	*P *= 0.001	*P *= 0.019	*P *= 0.844	*P *= 0.273	0.019	
						
Mouse genes						
Top 30% quantile	2631 ± 290	16190 ± 1828	7 ± 1	1214 ± 65	779 ± 136	6219 ± 794
versus						
bottom 30% quantile	8032 ± 2706	37391 ± 4615	8 ± 1	1450 ± 128	1496 ± 190	365 ± 16
	*P *= 0.001	*P *= 0.001	*P *= 0.444	*P *= 0.589	*P *= 0.001	

^a ^The human genes and the mouse genes are those analyzed in Figure 1. We used the Mann-Whitney U test to determine the significance of differences. For each case, we present the average value ± standard error of mean.

The weight ratio of a large tissue/organ to a small tissue/organ is much larger than the ratio in mRNA abundance required producing a significant difference in average intron length, total intron length and UTR length. However, large differences in tissue/organ weights do not produce significant differences in intron length or UTR length (Figure 1). This result is unexpected on the basis of the energetic cost hypothesis.

### Qualitatively estimating the energetic burden of long introns in highly expressed genes

We also qualitatively estimated the length and number of introns in genomes that may be selected against because of their energetic cost during transcription. In a highly expressed housekeeping gene (housekeeping genes are expressed in all cells in the human body, so their cumulative energetic burden is higher), let us assume that there is an intron with the threshold length (*L*) to trigger natural selection. Several studies have shown that most eukaryotic genes are expressed at the level of two or three copies of mRNA per cell [25-27], so a gene that produces 30 mRNA copies in each cell can be viewed as a highly expressed gene. The median half-life of human mRNA is about 10 h, and fast decay mRNAs have half-lives of < 2 h [28]. For a conservative estimation, we can assume that the gene needs to synthesize 30 mRNA copies every 2 h, that is, 360 mRNA copies per day, per cell. The expense of transcription is two ATP molecules per nucleotide. Therefore, transcription of the intron requires 360 × 2 *L *= 720 *L *ATP molecules per day in each cell. Estimates of the number of cells in an adult human body vary from 10^13 ^to 10^14 ^[29]. For a conservative estimation of the energetic cost of gene transcription, we used the higher value, 10^14 ^cells. As an adult human consumes about 200 mol of ATP per day [18,30], the energy consumption of each human cell is (200 × 6.02 × 10^23^)/10^14 ^= 1.2 × 10^12 ^ATP molecules per day. It should be noted that this is a conservative estimation; the energy consumption involved in strenuous exercises (for example, mountain climbing) may be as much as 10 times more than that used when resting [18]. The proportion of human daily energy consumption representing the energetic cost of the long putative intron of a highly expressed housekeeping gene (which can be considered as the coefficient of natural selection, *S*) is 720 *L*/(1.2 × 10^12^) = 6 *L *× 10^-10^. The recent effective population size (*Ne*) of humans is ≤ 10^4 ^[31,32]. According to *S *= 1/(2 *Ne*) as the margin above which natural selection is stronger than genetic drift, *L *= 1/(2 × 10^4^× 6 × 10^-10^) = 8.3 × 10^4 ^nt. In human genome, only 0.9% of introns are longer than this threshold. In principal, this estimation is applicable to the energetic cost of the transcription of a CDS or UTR.

The major differences between humans and mice are in their body sizes, their metabolic rates and their effective population sizes. We could not find an estimation of the number of cells in a mouse body. However we did find data on mass-specific metabolic rates [33,34], from which we can estimate energy consumption per mouse cell by assuming that human and mouse cells do not differ greatly in mass. The mass-specific metabolic rate of mice is 0.0151 W/g and that of humans is 0.00118 W/g [34], so a mouse cell uses ~12.8 times more energy than a human cell. As estimated above, the energy consumption of each human cell is about 1.2 × 10^12 ^ATP molecules per day, so that of each mouse cell is about 1.5 × 10^13 ^ATP molecules per day. The proportion of mouse daily energy consumption (*S*) representing the energetic cost of the long putative intron of a highly expressed housekeeping gene is (360 × 2 *L*)/(1.5 × 10^13^) = 4.8 *L *× 10^-11^, where *L *is defined as described in the previous paragraph. Different sources of data on the effective population size of mice are not consistent [35,36]; we retained a higher value (*Ne *= 8.1 × 10^5^) for a conservative estimation. Thus, in mice, the threshold length of introns to trigger natural selection is *L *= 1/(2 × 8.1 × 10^5^× 4.8 × 10^-11^) = 1.3 × 10^4 ^nt. Similar to the situation in humans, only a small fraction of introns in the mouse genome (6.8%) are longer than this threshold.

Owing to a lack of the required information (such as mRNA decay rates), it is impossible to accurately estimate the burden of long introns in other vertebrates and invertebrates. Considering that the effective population size of vertebrates is only about 10^4 ^[37], we suggest that long introns in highly expressed vertebrate genes are unlikely to be selected against. However, for invertebrates, with an effective population size of about 10^6 ^[37], it would be too bold to give a rough estimation.

Benefiting from the extensive studies on yeast *Saccharomyces cerevisiae*, we also found enough data to estimate the energetic burden of a long intron in a unicellular eukaryote. A gene that produces 30 mRNA copies in each cell can also be viewed as a highly expressed gene in yeasts [25-27]. The median half-life of yeast mRNAs is about 21 min, and the 90th percentile of mRNA half-lives is 10 min [26]. Conservatively, we assumed that such a gene would need to synthesize 30 mRNA copies every 10 min; that is, 30 × 24 × 60/10 = 4320 copies of mRNA every day. To transcribe a long intron, a yeast cell consumes 4320 × 2 *L *= 8340 *L *ATP molecules, where *L *is defined as previously. A yeast cell weighs 3.35 × 10^-11 ^g and the median value of yeast metabolic rates at eight different temperatures is 0.267 W/g [38], so the metabolic rate of a yeast cell is 8.9 × 10^-12 ^W, which can be convert to 1.39 × 10^13 ^ATP molecules per day. The proportion of yeast daily energy consumption representing the energetic cost of the putative long intron in a highly expressed gene is 8640 *L*/(1.39 × 10^13^) = 6.2 *L *× 10^-10^. The effective population size of yeasts is about 10^7 ^[37,39]. Thus, in yeasts, the threshold length of introns to trigger natural selection is *L *= 1/(2 × 10^7× ^6.2 × 10^-10^) = 81 nt. Unlike the situation in humans and mice, 86.5% of the introns in the genome of *S. cerevisiae *are longer than this threshold length. The fractional energetic cost of long introns may be overestimated here; thus the extant long introns, even in highly expressed genes, may be not under negative selection. At least, this result is helpful to explain the fact that unicellular eukaryotes generally have much shorter introns than mammals, and it is consistent with a previous study, which showed that energy is a constraint on evolutionary changes in yeast gene expression [39]. However, these estimations are at least seemingly contradictory to the observations that highly expressed genes have longer introns than weakly expressed genes in yeasts [40,41]. To reach a conclusion, further investigations are required.

Considered just from the point of view of the energetic cost of transcription, loss of entire introns may be favored in yeasts, but unlikely in mammals. On the other side, intron gain may be selected against in yeasts, but is most likely neutral, and thus, under genetic drift in mammals. This idea is consistent with the paucity of introns in yeast genes and the abundance of introns in animal genes [42,43]. Previously, the existence of different rates of intron loss in the evolution of different lineages was explained by differential retrotransposon activities [44-46]. We look forward to further evidence to determine whether selection to reduce energetic cost is a complementary explanation. In evolution, insertion of several nucleotides or various transposons into introns and deletion of short sequences from introns are much more frequent than gain and loss of entire introns. Considered just from the point of view of the energetic cost of transcription, the effects of common indels are negligible in mammals, but visible to natural selection in yeasts. This idea is similar to the theory of Lynch on the evolution of genome complexity [47,48].

### Alternate hypotheses for short introns in highly expressed genes

The first alternate hypothesis is the time cost hypothesis. RNA polymerase II can elongate only about 20–40 nt per second [1,49]. Recent evidence indicates that elongation, instead of RNA polymerase II recruitment, may be the predominant rate-limiting event in gene activation [50,51]. Therefore, gene length should have an important impact on the duration of gene expression. To be completely transcribed, a large gene in the human genome, such as *DMD *(2.3 Mb), requires 16 hours [49], a medium-sized gene (for example, *TUBE1*, 16.7 Kb) requires about 7–14 minutes, and a small gene (for example, *HBA2*, 834 bp) requires only about 20–40 seconds. Seoighe et al. [3] argued that the time required to transcribe multiple copies of mRNA is not a multiple of the transcription period of the first copy, because one template can be transcribed by several polymerases simultaneously [14]. Assuming a normal elongation rate of 0.03 seconds per nucleotide, the completion of the transcription of the first copy of a gene with *L *nt requires 0.03 *L *seconds. Assuming that there are *k *polymerases attached to the same template simultaneously, the completion of an additional copy of this transcript requires an additional 0.03 *L*/*k *seconds. Thus, the completion of the transcription of *n *copies of an mRNA requires *T*_*n *_= 0.03 *L *(1 + (*n*-1)/*k*) seconds. Apparently, if *n *<<*k*, *T*_*n*_≈ 0.03 *L*, gene length and transcript copy number are not related. However, in highly expressed genes, *n *is unlikely to be much smaller than *k*; thus, both gene length (*L*) and transcript copy number (*n*) contribute to the duration of transcription. To produce a large number of transcripts in a limited period of time, natural selection may decrease *L *or increase *k*. Unfortunately no genome-wide data on the values for *k *are now available in animals.

On the other side of the same coin, the time taken to transcribe introns has long been proposed to contribute to the timing mechanisms during development [52-54]. An extension of this hypothesis is that long introns may be maintained in some genes to reduce the number of mRNA products in the otherwise too-long time during which the genes are activated.

Another alternate hypothesis is that short genes may experience lower frequencies of abortive transcription and/or erroneous splicing than long genes. Successful transcription requires the polymerase to be stably associated with the DNA template during the elongation process. However, in some cases, the RNA-DNA duplex may not be stable enough to avoid abnormal pausing and arrest of elongation [55]. In a study of the human *DMD *gene, Tennyson et al. [49] found that 30–40% of transcription events were terminated or stopped at premature sites. Recently, Guenther et al. [50] found that many genes that have experienced transcription initiation do not produce complete transcripts. The short lengths of highly expressed genes may lead to a decreased possibility of a gene containing such sequences that are difficult to transcribe and cause abortion of elongation. In addition, evidence shows that long introns increase the frequency of erroneous splicing of nearby exons [56].

Long introns (and long UTRs) in highly expressed genes may also be selected against because of the crowding of active genes in a restricted interchromatin compartment [57].

A slightly more speculative and seemingly less likely hypothesis is that long introns are selected for in weakly expressed genes to avoid DNA damage resulting from transcriptional R-loops [6,58]. The fact that mRNA lengths have a similar correlation with expression levels as intron lengths [1,6,9] negates this hypothesis.

In addition, there is also the possibility that highly expressed genes are compact because their epigenetic regulation is relatively simple, as suggested by the "genome design" hypothesis [10]. Although there is some evidence against this idea, indicating that the lengths of intergenic spacers rather than those of introns are correlated with the complexity of epigenetic regulation [6,59], there is also evidence supporting it [60-64].

In contrast to the observations that highly expressed genes have short introns in animals, *P. patens *and the pollen of *A. thaliana*, highly expressed genes were found to have longer introns than weakly expressed genes in unicellular organisms, the sporophytes of *A. thaliana *and *Oryza sativa*, and, at least, the vegetative stage of the slime mould *Dictyostelium discoideum *[[40,41,65], Y.F. Huang and D.K. Niu, unpublished results from analyzing the data from [66]]. To date, there has been no satisfactory explanation for this difference [4,65]. Perhaps, the compact genomes and compact genes in large genomes have lost most of their nonfunctional sequences; thus, most of the retained intronic sequences have regulatory functions in gene expression [67-70]. Surprisingly, a weak, but significant negative correlation of mRNA length (and protein length) with expression level was found in all studied organisms [1,2,5,6,71-74], which is also generally explained by minimizing the energetic cost of gene expression. In light of this study, we suggest other potential reasons for the short introns of highly expressed genes: to minimize the duration of gene expression, or to reduce the frequencies of abortive transcription and/or erroneous splicing. However, we do not wish to completely discount the energetic cost hypothesis for mRNA compactness, because we have insufficient data on protein abundance (note that translation is also an expensive process).

## Conclusion

By assuming that intronic sequences are mostly junky, it is reasonable to attribute the fact that highly expressed genes have short introns to potential selection to minimize the energetic cost of gene expression. However, this hypothesis is not supported by our comparison of tissue/organ-specific genes between large tissue/organs and small tissue/organs in humans or mice. In addition, by conservatively selecting the values of a series of parameters, we quantitively estimated the energetic burden of a long intron in highly expressed genes. In mammals, the burden seems to be too negligible to trigger purifying selection against long introns. Further investigations are required to establish a new theory from a series of alternate hypotheses.

## Methods

The reference genomes of *Homo sapiens *(build 36, version 2) and *Mus musculus *(build 36, version 1) were downloaded from the NCBI genome database [75]. These genomes have been reviewed by NCBI staff. Genes with obvious annotation errors were excluded from our analyses. In the case of alternative splicing variants, we used the longest mRNA for analysis (although similar results were obtained by analyzing the shortest mRNA, data not shown). UTRs shorter than 30 nt were considered as trustless annotations. In analyzing UTR length, we retained only those genes with both 5' -UTRs and 3' -UTRs of 30 nt or longer. The UTR length of a gene is the sum of the lengths of its 5' UTR and 3' UTR.

The microarray gene expression datasets of *H. sapiens *and *M. musculus *were downloaded from GNF Genome Informatics Applications & Datasets [15,76]. These are the most extensive gene expression datasets freely available online. Besides quantitive signals, the datasets contain qualitative indicators of gene expression for each Affymetrix probe set in each tissue/organ sample: P (present), M (marginal), A (absent). Several probe sets may be annotated as one gene and each probe set has two repeats. In this study, we defined a gene as being expressed in a tissue/organ sample by a conservative criterion and a relaxed one. In the conservative criterion, all probe sets and repeats of a gene should be marked as P in the datasets, and in the relaxed criterion, two repeats of at least one probe set should be marked as P or M. These two criteria gave similar results. We present the results of analysis based on the conservative criterion in the main text of this paper, and those based on the relaxed criterion as Figure S1 and Table S1 of Additional File 3. Some probes of the probe sets annotated with a "_x" appended to the probe set name may cross-hybridize with other sequences, and so the resulting signal may partially arise from transcripts other than the one being intentionally measured (Affymetrix Technical Note, Array Design for the HGU133 set). We repeated our analysis by removing such probe sets from the gene expression datasets and obtained similar results (see Figure S2 and Table S2 of Additional File 3).

A greedy algorithm was used to match LTS genes and STS genes with similar expression levels. To maximize the number of gene pairs, the category with smaller gene number (STS genes in humans and LTS genes in mice) was used as the query set, and the category with larger gene number was used as the target set. For each gene in the query set, we selected the gene with the most similar expression level from the target set as the candidate target gene. If the within-pair difference was equal or smaller than the threshold of 20%, the query gene and the candidate target gene were viewed as a gene pair with similar expression levels. Adjusting this threshold to 10% gave similar results (Figure S3 and Table S3 of Additional File 3); a much lower threshold would result in too small a sample size to study. Similar to a previous study [6], the within-pair differences of expression levels was defined as

$$\left|\frac{A-B}{Max(A,B)}\right|$$

where A is the expression level of an LTS gene and B is the expression level of an STS gene. As shown in Figure S4 of Additional File 3, the within-pair differences in expression levels were not biased to either LTS genes or STS genes.

## Authors' contributions

D–KN and Y–FH conceived and designed the research. Y–FH performed the analysis. D–KN wrote the paper. Both authors read and approved the final manuscript.

## Supplementary Material

Additional file 1 A list of all the human tissue/organ-specific genes counted in Table 1. This list includes the gene symbols, gene features and some other details. Gene expression was defined by the conservative criterion described in the Methods and probe sets annotated with an "_x" appended to the probe set name were retained. Click here for file

Additional file 2 A list of all the mouse tissue/organ-specific genes counted in Table 1. This list includes the gene symbols, gene features and some other details. Gene expression was defined by the conservative criterion described in the Methods and probe sets annotated with an "_x" appended to the probe set name were retained. Click here for file

Additional file 3 Comparisons of compactness between LTS-STS gene pairs with similar expression levels and compactness between genes expressed at different levels. Figure S1 – Figure S3 present the results of the comparisons of LTS-STS gene pairs with similar expression levels selected based on criteria different from Figure 1. Table S1 – Table S3 show the results of the comparison of compactness between genes expressed at different levels. Click here for file
