# Supplementary material for: Evidence against the energetic cost hypothesis for the short introns in highly expressed genes
Source: BMC Evol Biol. 2008 May 20;8:154. doi: 10.1186/1471-2148-8-154 (PMC2424036; doi:10.1186/1471-2148-8-154)
Supplement: Additional file 3 — Comparisons of compactness between LTS-STS gene pairs with similar expression levels and compactness between genes expressed at different levels. Figure S1 – Figure S3 present the results of the comparisons of LTS-STS gene pairs with similar expression levels selected based on criteria different from Figure 1. Table S1 – Table S3 show the results of the comparison of compactness between genes expressed at different levels. [file 1471-2148-8-154-S3.doc]

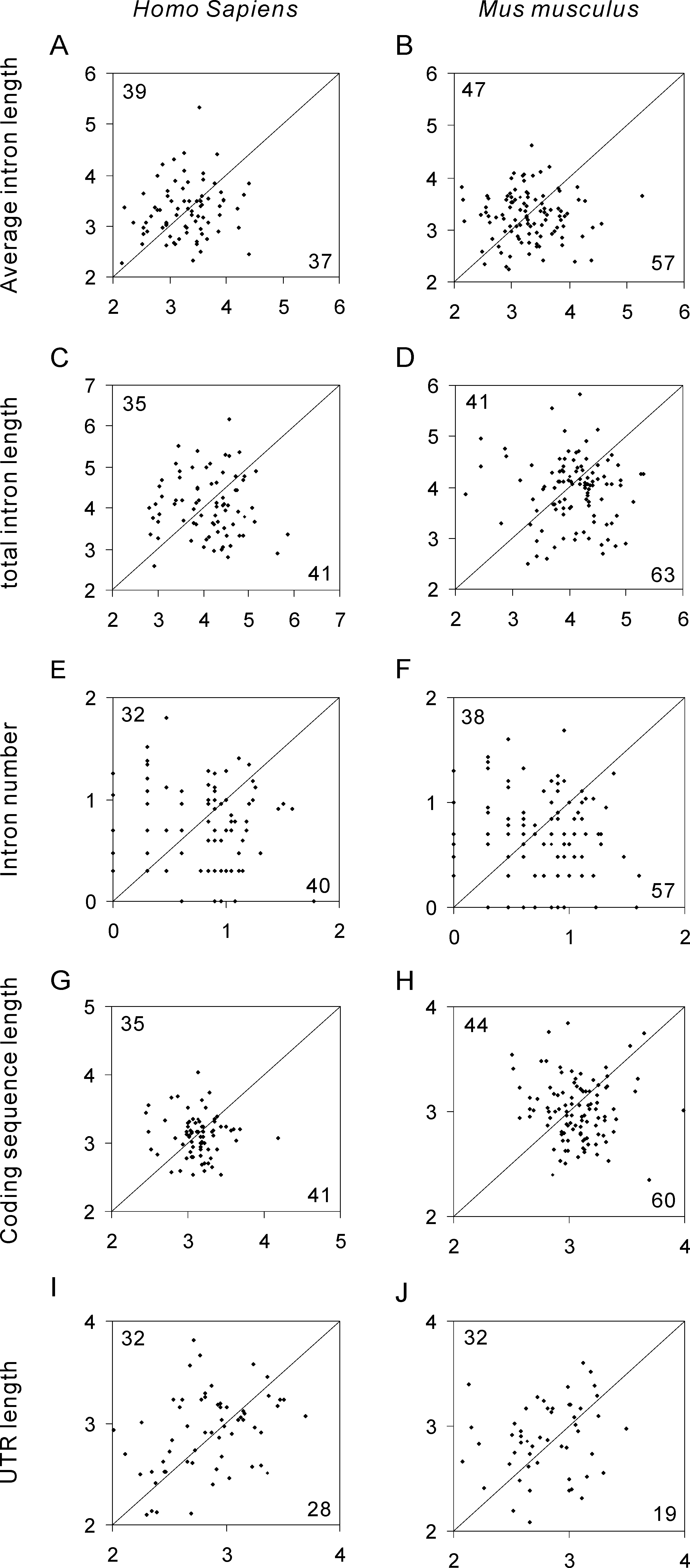


Figure S1. Comparison of large-tissue/organ-specific genes and small-tissue/organ-specific genes with similar expression levels. Gene expression was defined by a relaxed criterion, in which two repeats of at least one probe set should be marked as P or M. Probe sets annotated with an “_x” appended to the probe set name were retained. The upper limit of within-pair differences in expression level was set at 20%. The logarithm (base 10) values are shown. The Y axis represents small-tissue/organ-specific genes, while the X axis shows their large-tissue/organ-specific counterparts. Thus, the numbers of dots above (marked at the top left corner) and below (marked at the bottom right corner) the diagonal line illustrate the comparison between large-tissue/organ-specific genes and small-tissue/organ-specific genes. We performed Wilcoxon signed ranks tests to determine the significance of the differences. The number of gene pairs and the significance levels are: (A) 76, *P* = 0.73; (B) 104, *P* = 0.48; (C) 76, *P* = 0.68 ; (D) 104, *P* = 0.11; (E) 76, *P* = 0.33; (F) 104, *P* = 0.10; (G) 76, *P* = 0.43; (H) 104, *P* = 0.14; (I) 60, *P* = 0.51; (J) 51, *P* = 0.06.


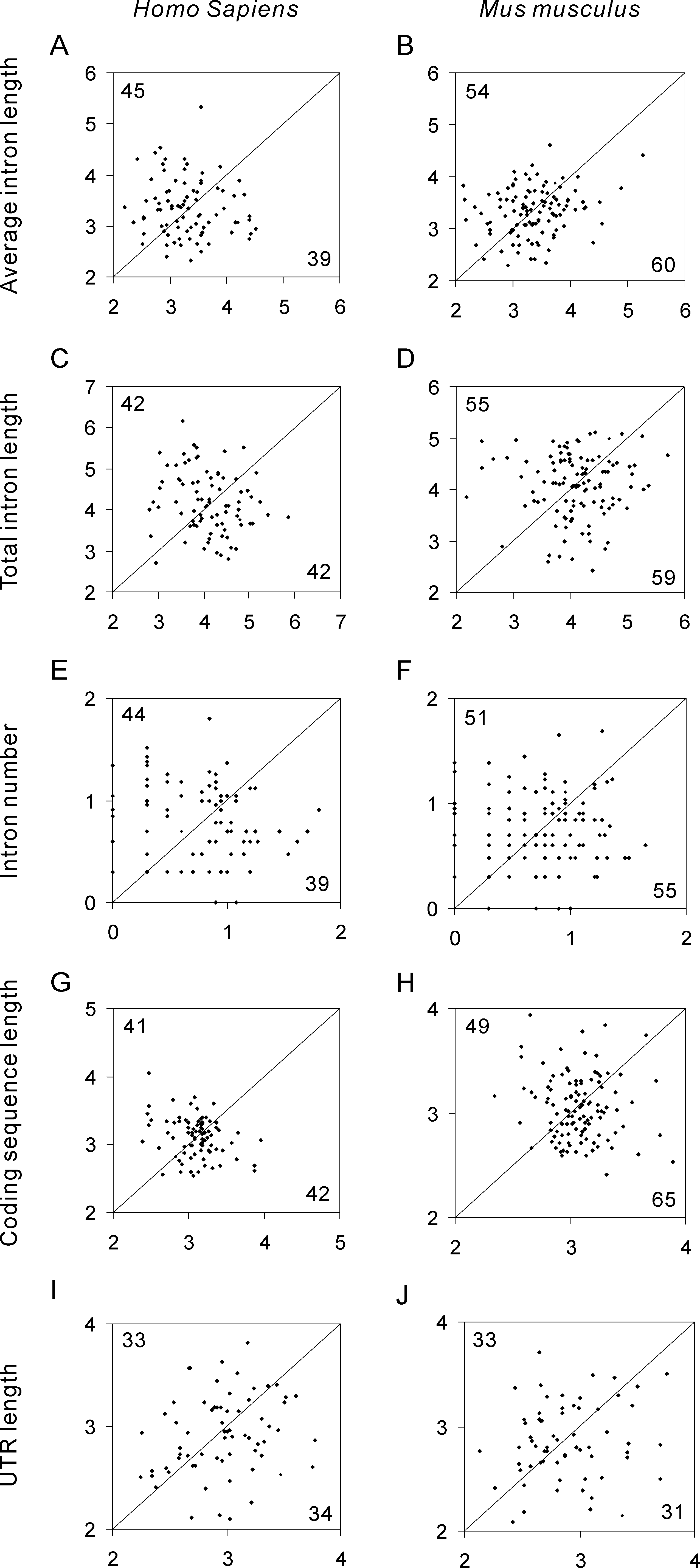


Figure S2. Comparison of large-tissue/organ-specific genes and small-tissue/organ-specific genes with similar expression levels. Probe sets annotated with an “_x” appended to the probe set name were removed. Gene expression was defined by a conservative criterion, in which all probe sets and repeats of a gene should be marked as P. The upper limit of within-pair differences in expression level was set at 20%. The logarithm (base 10) values are shown. The Y axis represents small-tissue/organ-specific genes, while the X axis shows their large-tissue/organ-specific counterparts. Thus, the numbers of dots above (marked at the top left corner) and below (marked at the bottom right corner) the diagonal line illustrate the comparison between large-tissue/organ-specific genes and small-tissue/organ-specific genes. We performed Wilcoxon signed ranks tests to determine the significance of the differences. The number of gene pairs and the significance levels are: (A) 84, *P* = 0.64; (B) 114, *P* = 0.40; (C) 84, *P* = 0.45; (D) 114, *P* = 0.998; (E) 84, *P* = 0.56; (F) 114, *P* = 0.72; (G) 84, *P* = 0.68; (H) 114, *P* = 0.74; (I) 67, *P* = 0.45; (J) 64, *P* = 0.86.


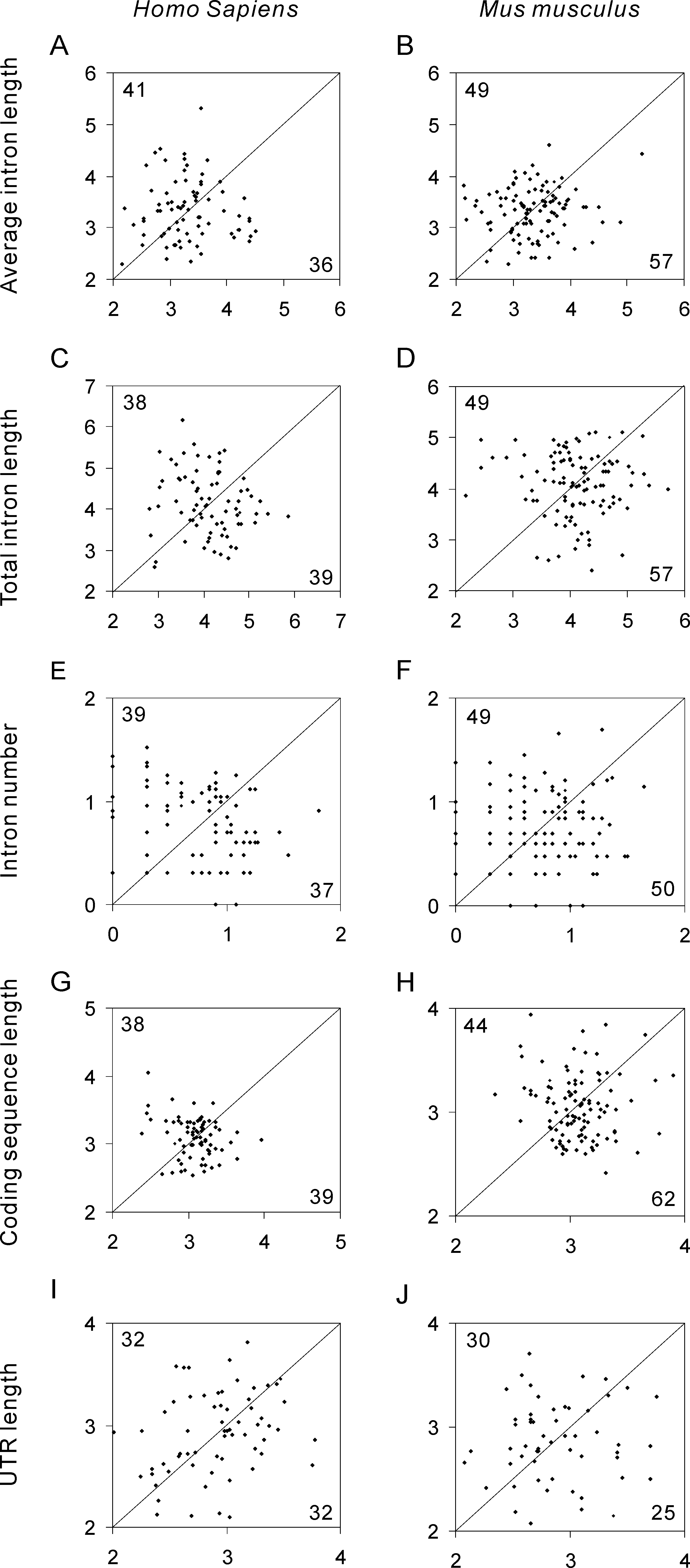


Figure S3. Comparison of large-tissue/organ-specific genes and small-tissue/organ-specific genes with similar expression levels. The upper limit of within-pair differences in expression level was set at 10%. Gene expression was defined by a conservative criterion. Probe sets annotated with an “_x” appended to the probe set name were retained. The logarithm (base 10) values are shown. The Y axis represents small-tissue/organ-specific genes, while the X axis shows their large-tissue/organ-specific counterparts. Thus, the numbers of dots above (marked at the top left corner) and below (marked at the bottom right corner) the diagonal line illustrate the comparison between large-tissue/organ-specific genes and small-tissue/organ-specific genes. We performed Wilcoxon signed ranks tests to determine the significance of the differences. The number of gene pairs and the significance levels are: (A) 77, *P* = 0.59; (B) 106, *P* = 0.49; (C) 77, *P* = 0.65; (D) 106, *P* = 0.92; (E) 77, *P* = 0.97; (F) 106, *P* = 0.78; (G) 77, *P* = 0.89; (H) 106, *P* = 0.61; (I) 64, *P* = 0.95; (J) 55, *P* = 0.99.


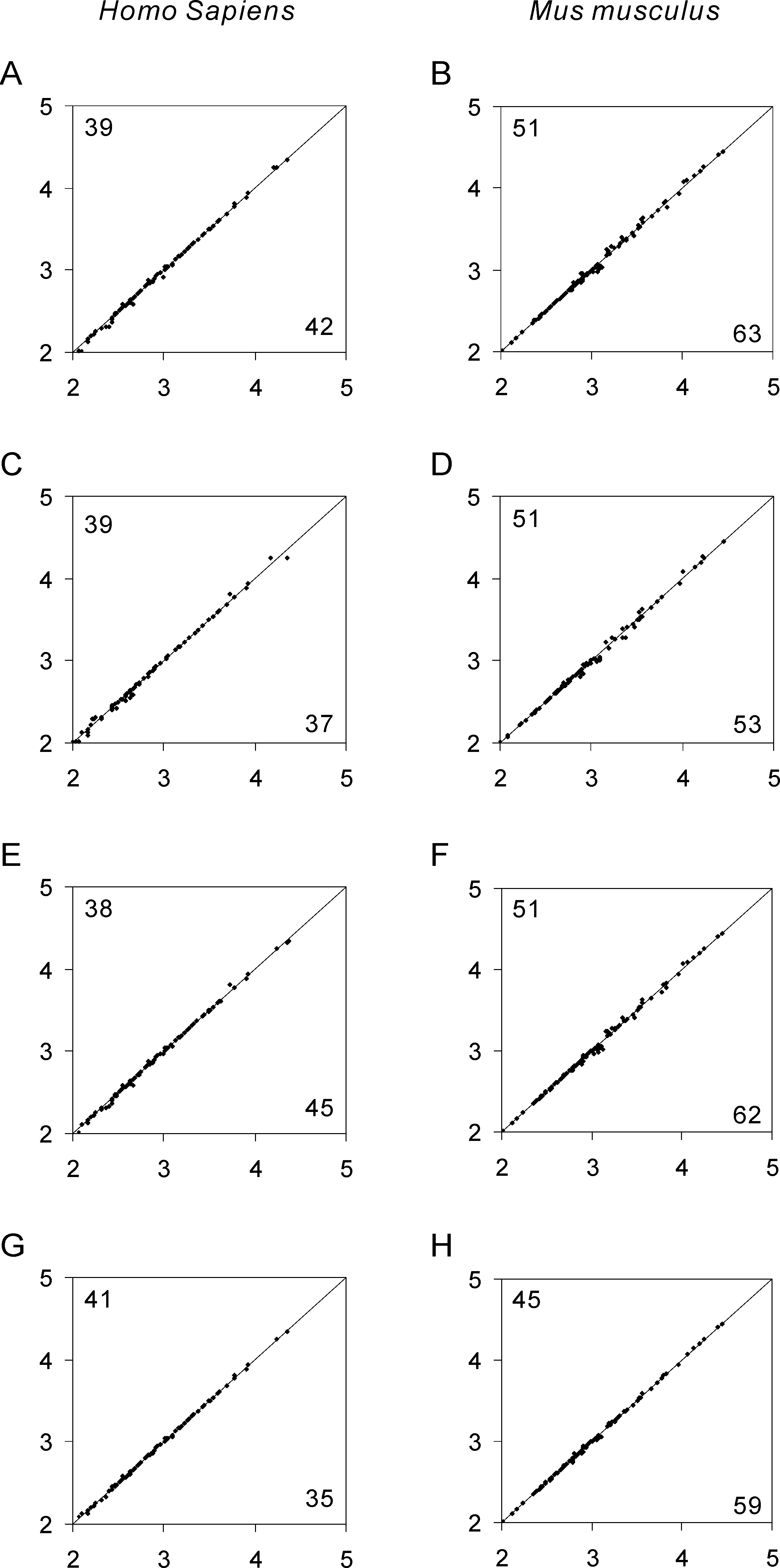


Figure S4. Comparison of the expression levels between large-tissue/organ-specific genes and small-tissue/organ-specific genes. The logarithm (base 10) values are shown. The Y axis represents small-tissue/organ-specific genes, while the X axis shows their large-tissue/organ-specific counterparts. Thus, the numbers of dots above (marked at the top left corner) and below (marked at the bottom right corner) the diagonal line illustrate the comparison between large-tissue/organ-specific genes and small-tissue/organ-specific genes. We performed Wilcoxon signed ranks tests to determine the significance of the differences. (A) 82 pairs of human genes analyzed in Figure 1, *P* = 0.601 ; (B) 116 pairs of mouse genes analyzed in Figure 1, *P* = 0.472; (C) 76 pairs of human genes analyzed in Figure S1, *P* = 0.608; (D) 104 pairs of mouse genes analyzed in Figure S1, *P* = 0.870; (E) 84 pairs of human genes analyzed in Figure S2, *P* = 0.190; (F) 114 pairs of mouse genes analyzed in Figure S2, *P* = 0.377. (G) 77 pairs of human genes analyzed in Figure S3, *P* = 0.643; (H) 106 pairs of mouse genes analyzed in Figure S3, *P* = 0.164.

Table S1. Comparison of compactness between genes expressed at different levels^a^

|  | Average intron length | Total intron length | Intron number | CDS length | UTR length | Expression level |
| --- | --- | --- | --- | --- | --- | --- |
| Human genes | |  |  |  |  |  |
| Top 30% quantile | 2369 ± 570 | 21374 ± 5476 | 8 ± 1 | 1255 ± 101 | 594 ± 74 | 4626 ± 706 |
| versus |  |  |  |  |  |  |
| bottom 30% quantile | 8876 ± 4529 | 86240 ± 35694 | 10 ± 2 | 2032 ± 326 | 1450 ± 164 | 170 ± 11 |
|  | *P* = 0.001 | *P* = 0.025 | *P* = 0.966 | *P* = 0.015 | *P* < 0.001 |  |
|  |  |  |  |  |  |  |
| Mouse genes | |  |  |  |  |  |
| Top 30% quantile | 2680 ± 391 | 15021 ± 2594 | 6 ± 1 | 1159 ± 62 | 631 ± 87 | 5864 ± 811 |
| versus |  |  |  |  |  |  |
| bottom 30% quantile | 7684 ± 3062 | 43151 ± 12571 | 9 ± 1 | 1451 ± 136 | 1222 ± 131 | 277 ± 15 |
|  | *P* = 0.042 | *P* = 0.003 | *P* = 0.185 | *P* = 0.564 | *P* < 0.001 |  |

^a^ The human and mouse genes are those analyzed in Figure S1. We used the Mann-Whitney U test to determine the significance of differences. For each case, we present the average value ± standard error of the mean.

Table S2. Comparison of compactness between genes expressed at different levels^a^

|  | Average intron length | Total intron length | Intron number | CDS length | UTR length | Expression level |
| --- | --- | --- | --- | --- | --- | --- |
| Human genes | |  |  |  |  |  |
| Top 30% quantile | 2599 ± 540 | 27649 ± 7229 | 9 ± 1 | 1444 ± 149 | 928 ± 126 | 5462 ± 846 |
| versus |  |  |  |  |  |  |
| bottom 30% quantile | 10869 ± 4231 | 92771 ± 33138 | 9 ± 1 | 1745 ± 238 | 1525 ± 241 | 255 ± 12 |
|  | *P* < 0.001 | *P* = 0.008 | *P* = 0.606 | *P* = 0.629 | *P* = 0.070 |  |
|  |  |  |  |  |  |  |
| Mouse genes | |  |  |  |  |  |
| Top 30% quantile | 2655 ± 294 | 16246 ± 1866 | 7 ± 1 | 1215 ± 66 | 684 ± 78 | 6366 ± 811 |
| versus |  |  |  |  |  |  |
| bottom 30% quantile | 8226 ± 2782 | 38221 ± 4714 | 8 ± 1 | 1427 ± 126 | 1510 ± 198 | 359 ± 16 |
|  | *P* = 0.001 | *P* < 0.001 | *P* = 0.438 | *P* = 0.583 | *P* = 0.001 |  |

^a^ The human and mouse genes are those analyzed in Figure S2. We used the Mann-Whitney U test to determine the significance of differences. For each case, we present the average value ± standard error of the mean.

Table S3. Comparison of compactness between genes expressed at different levels^a^

|  | Average intron length | Total intron length | Intron number | CDS length | UTR length | Expression level |
| --- | --- | --- | --- | --- | --- | --- |
| Human genes | | | | | | |
| Top 30% quantile | 2888 ± 658 | 29954 ± 7931 | 8 ± 1 | 1323 ± 97 | 771 ± 117 | 5036 ± 744 |
| versus |  |  |  |  |  |  |
| bottom 30% quantile | 11516 ± 4601 | 96067 ± 35650 | 9 ± 1 | 1785 ± 251 | 1672 ± 246 | 264 ± 14 |
|  | *P* = 0.001 | *P* = 0.015 | 0.661 | 0.333 | 0.002 |  |
|  |  |  |  |  |  |  |
| Mouse genes | | | | | | |
| Top 30% quantile | 2584 ± 303 | 16270 ± 2152 | 7 ± 1 | 1188 ± 97 | 787 ± 164 | 6106 ±864 |
| versus |  |  |  |  |  |  |
| bottom 30% quantile | 8206 ± 2950 | 39319 ± 4974 | 9 ± 1 | 1454 ± 133 | 1525 ± 209 | 345 ± 15 |
|  | *P* = 0.001 | *P* < 0.001 | *P* = 0.320 | *P* = 0.178 | *P* = 0.002 |  |

^a^ The human and mouse genes are those analyzed in Figure S3. We used the Mann-Whitney U test to determine the significance of differences. For each case, we present the average value ± standard error of the mean.
